# The ATP-Releasing Maxi-Cl Channel: Its Identity, Molecular Partners, and Physiological/Pathophysiological Implications

**DOI:** 10.3390/life11060509

**Published:** 2021-05-31

**Authors:** Ravshan Z. Sabirov, Md. Rafiqul Islam, Toshiaki Okada, Petr G. Merzlyak, Ranokhon S. Kurbannazarova, Nargiza A. Tsiferova, Yasunobu Okada

**Affiliations:** 1 Division of Cell Signaling, National Institute for Physiological Sciences (NIPS), Okazaki 444-8787, Japan; rafiq.bmb_du@yahoo.com (M.R.I.); okada@veneno.co.jp (T.O.); galores@list.ru (P.G.M.); rano_1971@mail.ru (R.S.K.); ntsiferova@mail.ru (N.A.T.); 2 Institute of Biophysics and Biochemistry, National University of Uzbekistan, Tashkent 100174, Uzbekistan; 3 Department of Biochemistry and Molecular Biology, Jagannath University, Dhaka 1100, Bangladesh; 4 Veneno Technologies Co. Ltd., Tsukuba 305-0031, Japan; 5 Department of Physiology, Kyoto Prefectural University of Medicine, Kyoto 602-8566, Japan; 6 Department of Physiology, School of Medicine, Aichi Medical University, Nagakute 480-1195, Japan

**Keywords:** maxi-anion channels, Maxi-Cl, biophysical properties, purinergic signaling, ATP release, glutamate release, SLCO2A1, ANXA2, S100A10, diseases

## Abstract

The Maxi-Cl phenotype accounts for the majority (app. 60%) of reports on the large-conductance maxi-anion channels (MACs) and has been detected in almost every type of cell, including placenta, endothelium, lymphocyte, cardiac myocyte, neuron, and glial cells, and in cells originating from humans to frogs. A unitary conductance of 300–400 pS, linear current-to-voltage relationship, relatively high anion-to-cation selectivity, bell-shaped voltage dependency, and sensitivity to extracellular gadolinium are biophysical and pharmacological hallmarks of the Maxi-Cl channel. Its identification as a complex with SLCO2A1 as a core pore-forming component and two auxiliary regulatory proteins, annexin A2 and S100A10 (p11), explains the activation mechanism as Tyr23 dephosphorylation at ANXA2 in parallel with calcium binding at S100A10. In the resting state, SLCO2A1 functions as a prostaglandin transporter whereas upon activation it turns to an anion channel. As an efficient pathway for chloride, Maxi-Cl is implicated in a number of physiologically and pathophysiologically important processes, such as cell volume regulation, fluid secretion, apoptosis, and charge transfer. Maxi-Cl is permeable for ATP and other small signaling molecules serving as an electrogenic pathway in cell-to-cell signal transduction. Mutations at the *SLCO2A1* gene cause inherited bone and gut pathologies and malignancies, signifying the Maxi-Cl channel as a perspective pharmacological target.

## 1. Introduction

ATP is produced and consumed at high rates inside cells. The resting intracellular ATP concentration is about 1.3 mM in cultured HeLa cells [1], which is close to the ATP content in other cultured cell lines, such as MIN6 (~1 mM) and COSm6 (~1.4 mM) [2,3]. Cytosolic ATP is not constant and may change drastically and rapidly (several folds within minutes) upon stimulation, such as apoptosis induction or Ca^2+^ influx [1,4]. The extracellular space normally contains very small amounts of ATP (1 to 10 nM) under physiological conditions [5,6], which is continuously cleared off by different ectonucleoside triphosphate diphosphohydrolases (NTPDases including CD39), which hydrolyze ATP and ADP to AMP that is degraded to adenosine by 5′-ectonucleotidase CD73 [7,8,9]. Although some amount of extracellular ATP can be recovered by ectoadenylate and ectonucleoside kinases, most of it is released from the cytosol either upon cell lysis or by more specific mechanisms involving exocytosis, activity of transporters, and electrogenic conductive release [10,11,12,13,14,15,16,17,18,19,20,21,22,23,24].

In biological fluids, ATP exists as a polyvalent organic anion, possessing from one to four negative charges depending on pH and binding to Mg^2+^ (see Table 1 in [13]). The effective radius of ATP^4–^ and MgATP^2–^ is about 0.6 nm (see Table 2 in [13]). Therefore, if an ion channel has a pore with poor or, preferably, anionic selectivity and the size is large enough to provide physical passage of the nucleotide, it could serve as an electrogenic pathway for ATP. It should be noted that a large concentration difference between cytosol and external spaces, together with a negative potential inside the cells, provides a very favorable outwardly directed electrochemical potential gradient for conductive ATP release. The concept of an “ATP release channel” has been validated for two groups of ion channels: (i) non-selective cation channels, including connexin and pannexin hemichannels [25,26,27,28], P2 × 7 [29,30], calcium homeostasis modulator CALHM1 [19,22,31], and Piezo1 [32] and (ii) CFTR and volume-regulated anion channels (VRACs), including the volume-sensitive outwardly rectifying anion channel (VSOR) and the maxi-anion channel (MAC) [13,18,22,23,33,34,35,36]. Acknowledging the contributions of every mentioned conductive pathway in the regulated ATP release, here we shall focus on the maxi-anion channels.

After invention of the patch-clamp technique, it was very surprising to see that ion channels differ so greatly in their unitary amplitude, which may range from a fraction of picosiemens for ClC family chloride channels [37], up to several hundreds of picosiemens for MACs, or even to a nanosiemen level for bacterial mechanosensitive channels, MscS and MscL [38]. Obviously, large-conductance channels can accomplish their biological functions using just a few channel molecules, whereas tiny-conductance ones allow fine tuning of cellular responses, although at the expense of synthesizing more channel proteins. Perhaps, the MACs with a unitary amplitude of 200–500 pS are close to the upper conductance limit for mammalian plasma membrane ion channels.

The first available report on the anion channels of 430 pS in cultured muscle cells was published by Blatz and Magleby in 1983 [39]. Similar large-conductance anion channels were observed later in almost every type of cell, including placenta, endothelium, lymphocyte, cardiac myocyte, neuron, and glial cells [14,18,33,40,41,42,43,44], as well as cells from different species including trout gills [45], frog muscles and nerves [46,47], sheep and bovine gastrointestinal tracts, rumen and omasum [48,49,50,51], porcine [52], and bovine [53] endothelium, indicating that MACs are preserved throughout the evolution. In the present review, we shall briefly outline the main properties of MACs and focus on the molecular identity and partners.

## 2. Phenotype and Biophysical Profile of Maxi-Cl

**Figure 1:**
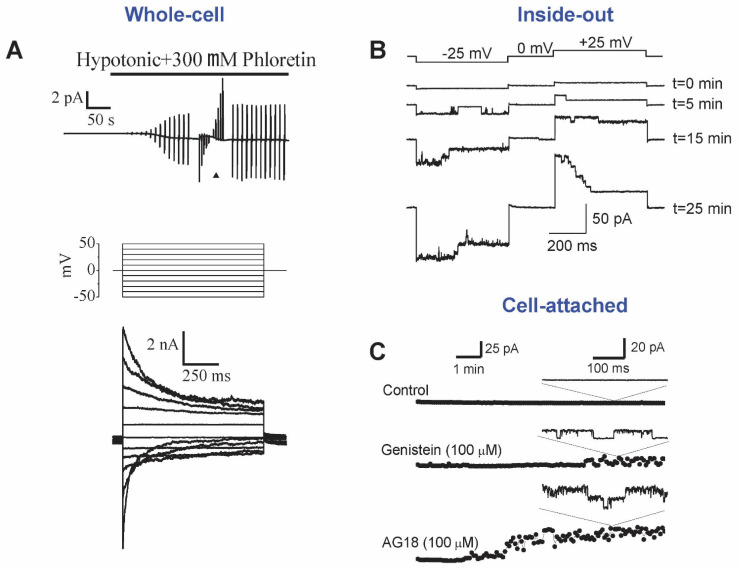
Maxi-Cl activation in mammary C127 cells at the macroscopic current and single-channel levels. (**A**) Whole-cell currents activated in response to hypoosmotic stress; pipette ATP was omitted to favor dephosphorylation and phloretin was used to block residual VSOR currents. (**B**) Consecutive activation of single Maxi-Cl channels upon patch excision into an artificial intracellular solution. (**C**) Maxi-Cl activation in the on-cell mode in response to pharmacological inhibition of the tyrosine kinases. Adopted from [54,62].

**Figure 2:**
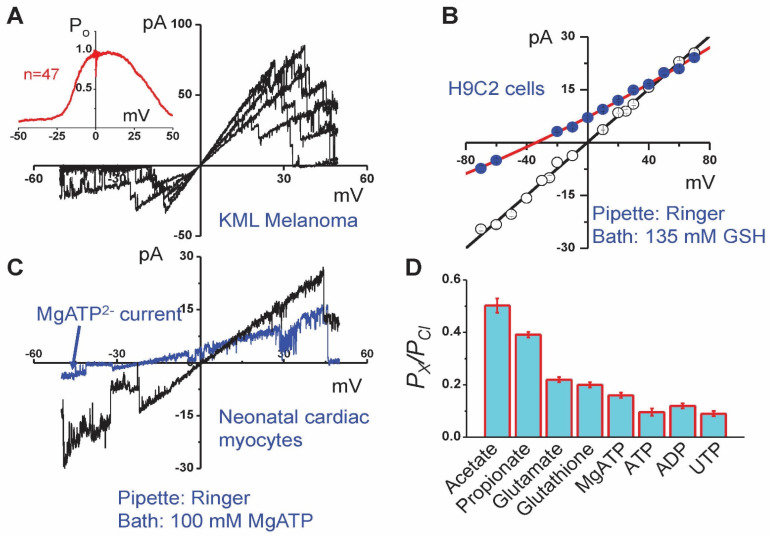
Maxi-Cl voltage dependence and permeability to physiologically important organic anions. (**A**) Six consecutive traces of the current response to the ramp pulse applied to KML melanoma cells. Inset shows the open-channel probability obtained by averaging 47 traces recorded from the same patch. Experimental conditions were the same as reported previously [54]. (**B**) Current-to-voltage relationship of the Maxi-Cl excised from H9C2 cells into the normal Ringer solution (open symbols) and after replacing the bath with a solution containing 135 mM GSH and 11 mM Cl (filled blue symbols); the curve reverses at –34.8 ± 0.9 mV, yielding the *P_GSH_/P_Cl_* = 0.20 ± 0.01. Experimental conditions were the same as reported previously [54,63,71]. (**C**) The current responses to the ramp pulse in normal Ringer solution (black) and after replacing the bath with a solution containing only 100 mM MgATP (blue) in neonatal cardiac myocytes. The small inward current was carried by MgATP^2–^. (**D**) Maxi-Cl permeability to short chain fatty acids and small signaling molecules evaluated in C127 cells and neonatal rat cardiomyocytes. Adopted from [13,57,67].

Single-channel events with a large conductance of over 200 pS and anionic selectivity are usually referred to as maxi-anion channels. However, depending on cell type and physiological or pathophysiological context, the amplitude, selectivity, voltage dependence, activation stimuli, and regulation by intracellular signaling cascades greatly differ. When MAC events were compared under the same experimental conditions (activation by patch excision and recording in the symmetrical inside-out configuration with normal Ringer solution, both in pipette and bath), we observed its fairly uniform phenotype characterized with a unitary conductance of 300–400 pS, linear and symmetrical current-to-voltage relationship, relatively high anion-to-cation selectivity with *P_Cl_/P_cation_* of >8, bell-shaped voltage dependency with maximal open-channel probability at around 0 mV and half-maximal closing at about 20–30 mV, and sensitivity to extracellular Gd^3+^ ions. We designated this phenotype as Maxi-Cl [33]. Figure 1 and Figure 2 illustrate the examples of Maxi-Cl activity recorded at the macroscopic level in the whole-cell mode as well as at the single-channel level, both in cell-attached and inside-out modes. Normally, intracellular pipette solutions contain millimolar amounts of ATP, which keeps the Maxi-Cl in the closed state. However, when metabolic deprivation is achieved by omitting pipette ATP in the whole-cell mode, large macroscopic Maxi-Cl currents with characteristic inactivation at moderate positive and negative voltages can be observed (Figure 1A). Membrane excision into an ATP-free intracellular-like medium favors Maxi-Cl activation (Figure 1B). When tyrosine phosphorylation is suppressed by specific inhibitors, Maxi-Cl channels become activated even at normal intracellular ATP content, as it is shown in Figure 1C. In excised inside-out patches, Maxi-Cl is fully active at around 0 mV and readily closes at more positive or negative potentials (Figure 2A). In the open state, Maxi-Cl is permeable to glutathione (Figure 2B), MgATP (Figure 2C), to short-chain fatty acids, and to nucleotide di- and triphosphates (Figure 2D).

MACs of the Maxi-Cl phenotype were observed in cells of different origins, such as mammary C127 cells [54], L929 fibrosarcoma cells [55], kidney macula densa cells [56], primary cultured neonatal and adult cardiomyocytes [57,58], primary cultured astrocytes [59,60], embryonic and adult fibroblasts [61,62], and freshly isolated thymic lymphocytes [63], as well as murine KML melanoma (Figure 2A) and rat heart myoblastic H9C2 cells (Figure 2B). The Maxi-Cl phenotype accounts for about 60% of the reports hitherto published on MACs [33].

Although a large amplitude is the phenotypical hallmark of Maxi-Cl, many researchers have described substates with conductances ranging from 15 to 200 pS [53,57,64,65,66]. We observed a substate of ~200 pS in cultured cardiomyocytes that was favored by hypoxia [57], suggesting that the cytosolic redox system could govern the channel transition between different substates.

**Figure 3:**
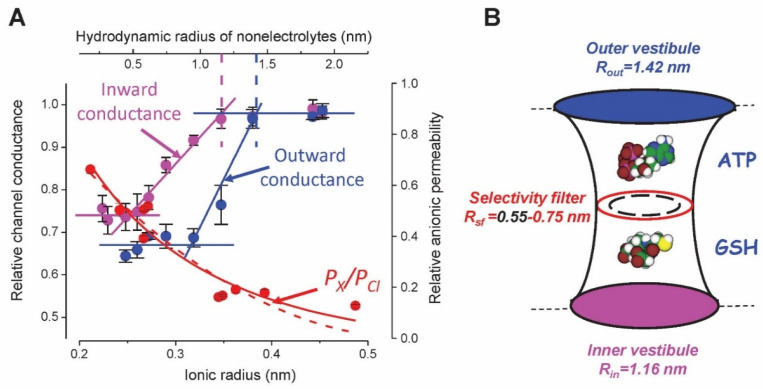
Geometry of the Maxi-Cl channel pore probed with ion permeability and nonelectrolyte partitioning. (**A**) Permeability to organic anions of different sizes (red symbols and curves) and relative channel conductance in the presence of nonelectrolytes (ethylene glycol, glycerol, and polyethylene glycols 200–4000) applied from the intracellular (magenta symbols and lines) and extracellular (blue symbols and lines) side of the membrane patch. The red dashed and solid lines are approximations of permeability using the excluded area theory without or with taking into account the friction force yielding the pore radii of 0.55 nm and 0.75 nm, respectively. The vertical dashed lines in magenta and blue mark the radii of the inner and outer vestibules, respectively. Adopted from [67,72]. (**B**) Maxi-Cl pore geometry pictured using estimates in (**A**). The molecules of ATP and GSH were drawn using Molecular Modeling Pro software (Norgwyn Montgomery Software Inc.) and are shown approximately in scale.

The pore of Maxi-Cl is fairly wide and slightly asymmetric, with a more spacious extracellular vestibule with the radius (*R*) of ~1.42 nm and a narrower intracellular vestibule with *R* ~1.16 nm, as estimated by the nonelectrolyte partitioning method [67] (Figure 3A)**.** The radius of the selectivity filter deduced from the cut-off size of permeable organic anions was found to be 0.33–0.36 nm in L6 myoblasts [68] and vascular smooth muscle cells [69]. However, fast voltage-dependent open-channel blockage by ATP in mammary C127 cells with the binding site around the median part would suggest a much wider path for anions. Indeed, fitting of the size dependence of the Maxi-Cl permeability to organic anions by excluded area theory equations yielded *R* of 0.75 nm when frictional forces were taken into account [67] (Figure 3A). This value is in agreement with the appreciable permeability of Maxi-Cl to ATP^4–^, with permeability ratio *P_ATP_/P_Cl_* of ~0.1 in C127 cells, cardiomyocytes, astrocytes, and macula densa [54,56,57,58,60,70]. The Maxi-Cl channel was found to be permeable to MgATP^2^^−^ (*P_MgATP_/P_Cl_* ~0.16) in cardiomyocytes [57], and to ADP^3^^−^ (*P_ADP_/P_Cl_* = 0.12) and UTP^4^^−^ (*P_UTP_/P_Cl_* = 0.09) in C127 cells [13] (see Figure 2D), all of them having calculated effective radii of ~0.53–0.61 nm [13]. The relative dimensions of the channel pore and permeating signaling molecules, ATP and glutathione (GSH, see discussion hereinafter) are illustrated in Figure 3B. Small but measurable permeability of the Maxi-Cl pore to nucleotides provides the physical basis for its physiological function as a pathway for electrogenic nucleotide release in purinergic cell-to-cell signaling.

## 3. Activation Stimuli of Maxi-Cl

Even in Maxi-Cl-rich cells, cell-attached patches are usually silent with almost no channel activity in the resting state. Osmotic swelling was the first stimulus shown to activate Maxi-Cl in mouse N1E115 neuroblastoma cells [73]. Later, a similar effect was demonstrated in primary cultured astrocytes [59,74], rabbit cortical collecting duct RCCT-28 cells [75], mammary C127 cells [54,70], and cardiomyocytes [57,58]. Activation in kidney macula densa cells by salt stress [56] could also be attributed to a volume increase since these cells profoundly swell in response to high NaCl in the tubular perfusate due to massive NaCl entry via a furosemide-sensitive pathway [76,77]. Volume sensitivity could be due to mechanosensing because Maxi-Cl can also be activated by negative hydrostatic pressure in silent inside-out patches [75]. Since Maxi-Cl can be activated by the Ca-ionophore A23187 [47,52,78,79], volume sensitivity could also be mediated by a swelling-induced intracellular Ca^2+^ increase [80]. A number of other stimuli have been reported to activate MACs, including application of endothelin-1 [81], bombesin [78], bradykinin [52], agonists of the A1-adenosine receptor [82] and the NK-1 receptor [66,83], antiestrogens [84,85], and heat [86,87]. In addition to the Ca sensitivity, the activation mechanism may involve G-protein signaling, as evidenced by activation or inactivation by GTPγS depending on the cellular context [66,82,88,89,90]. A role of cytoskeleton in the activation mechanism has also been proposed [75].

In a detailed study of the activation mechanisms of Maxi-Cl in Maxi-Cl-rich mammary C127 cells, Toychiev et al. [62] demonstrated that MgATP, but not its non-hydrolysable analog, Mg-AMP-PNP, completely abolished channel activation upon patch excision with *IC_50_* of ~29 µM. Tyrosine kinase inhibitors cancelled the MgATP-induced inhibition in excised patches and were able to activate the channel in an on-cell mode (see Figure 1C). Inhibitors of tyrosine but not serine/threonine phosphatases effectively blocked channel activation. In mice deficient in receptor protein tyrosine phosphatase ζ (RPTP ζ), the Maxi-Cl activity was about half of that in WT animals, and reintroducing this phosphatase restored the diminished channel activation in primary fibroblasts [62]. Consistent with these findings, dephosphorylation of Tyr23 at the regulatory component of Maxi-Cl, ANXA2, governs the channel activation [91]. In excised patches, the Maxi-Cl activity was enhanced by free Ca^2+^ ions with *K_d_* of ~0.5 µM in a manner dependent on the S100A10 expression [91], thus providing a mechanistic clue for earlier reports on Ca^2+^-dependent functioning of this channel [47,52,78,79].

## 4. Molecular Identity of Maxi-Cl: Rejected Candidates

Mitochondrial outer membranes contain large amounts of the mitochondrial porin termed the voltage-dependent anion channel (VDAC) [92,93]. When reconstituted into the artificial lipid bilayers, it forms ion channels that are phenotypically reminiscent of the maxi-anion channels: they have large conductance of app. 500 pS in 100 mM KCl [94,95], weak anion selectivity, and voltage-dependent gating similar to that of MACs with high open probability at 0 mV and transition to a less conductive state at positive and negative voltages [96,97]. It was important that the VDAC channel had a wide nano-sized pore with blockage by and permeability to ATP [98,99,100]. Therefore, it was natural to suppose that this protein somehow reaches the plasma membrane and functions there as the maxi-anion channel [57,84,101,102,103,104,105,106]. This idea was supported by biochemical isolation of the VDAC protein from preparations of plasma membranes of different origins and by finding of the genetic mechanism of generation of the plasmalemmally expressed VDAC protein (pl-VDAC) through its targeting of the plasma membrane with a signal peptide encoded by an alternative first exon [102]. The signal peptide is eventually cleaved away, yielding the plasmalemmal VDAC identical to the mitochondrial protein. We have indeed found the presence of mRNA coding for pl-VDAC in the mammary C127 cells (Sabirov and Okada, unpublished observation) confirming results obtained in mouse NIH3T3 fibroblasts [103], C1300 neuroblastoma cells [104], and fibroblasts from wild-type, but not *vdac1−/−,* mice [57]. However, the Maxi-Cl activity was recorded in fibroblasts isolated from mice with deletion of all individual *vdac1*, *vdac2,* and *vdac3* genes encoding the three isoforms of the VDAC protein, as well as in cells isolated from *vdac1/vdac3* double-deficient knock-out animals [61]. These results, together with the unaltered presence of Maxi-Cl in cells from *vdac2*-silenced *vdac1/vdac3* double-deficient cells, argued against the “pl-VDAC = Maxi-Cl” hypothesis. When we overexpressed the pl-VDAC gene (kindly provided by Dr. R. Buettner) in HEK293T cells, we did not observe the Maxi-Cl phenotype, although noisy low-amplitude events could be seen upon patch excision (see Figure 1 in [72]). Comparison of the biophysical profile of Maxi-Cl and VDAC determined under the same ionic environment suggests that there are significant differences in key pore properties, such as chloride dependence of the channel amplitude, which is linear for VDAC but saturates for Maxi-Cl, and discrimination between cations and anions under 100/1000 mM KCl gradient, which is substantial for Maxi-Cl (*P_Cl_/P_K_ =* 13.5) and virtually absent for VDAC under the same conditions (see Table 1 in [72]). Certainly, this result does not deny the very expression of the VDAC protein on the plasmalemma, where it could function as a receptor for plasminogen kringle 5 and for neuroactive steroids, or as an NADH/NADPH-dependent ferricyanide reductase (see for review [72]).

Pannexin and connexin hemichannels have been implicated in the conductive ATP release [25,26,27,28]. It could happen that some of these proteins function as MACs. However, the Maxi-Cl activity in L929 fibrosarcoma cells was insensitive to blocking peptides specific to pannexin 1 (^10^Panx1) and connexin 43 (Gap27), as well as to siRNAs targeting pannexins 1 and 2 and connexin 43 [55]. Thus, it was concluded that Maxi-Cl and hemichannels are separate pathways for anions including nucleotides.

Gene *tweety* in *Drosophila flightless* locus encodes an ion channel-like protein with five or six transmembrane domains and a partially hydrophobic P-region-like fragment. The human homologs of tweety, hTTYH1–3, were suggested to function as Ca^2+^-activated maxi-anion channels [107,108]. However, we failed to record the Maxi-Cl channel activity upon overexpression of two splice-variants of this gene, TTYH1-E and TTYH1-SV (kindly provided by Dr. M. Suzuki) in HEK293T cells, which lack endogenous Maxi-Cl activity [109]. Thus, the “hTTYH = Maxi-Cl” hypothesis should be rejected, although the “hTTYH = some other MAC” possibility still remains and should be verified.

Based on preliminary proteomics studies of membrane preparations from Maxi-Cl-rich C127 cells, we tested the effects of silencing of the following genes on the channel activity in these cells, and all were excluded from the candidates: (1) *Tmem* family members 63a [110] and 62, 65, 97, 138, 167b, and 189 [111]; (2) *Slc* family members *35d2* [110] and 15a4, 25a11, 25a3, 25a4, 25a5, 2a3, 33a1, 44a1, and 44a2 [111]; and (3) Annexin family members 1, 3, and 11 [111]. Involvements of *Praf2*, *Tm4sf1,* and Ttyh2 were also excluded based on heterologous expression in Maxi-Cl-deficient cells [111].

## 5. Molecular Identity of Maxi-Cl: The Pore Component

**Figure 4:**
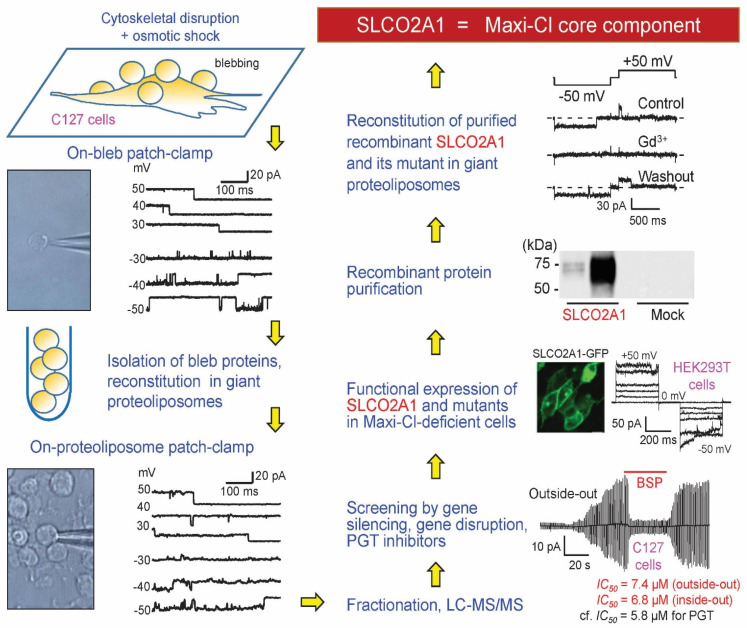
Steps in molecular identification of Maxi-Cl. The scheme was modelled after [35,113]. See text for details.

After failing with numerous probable candidates, we decided to start ab initio Maxi-Cl channel identification (Figure 4). First, we noticed that membrane blebs formed upon osmotic swelling of C127 cells with disrupted cytoskeletons exhibited high Maxi-Cl currents, which were in the constitutively active state and did not need to be activated by patch excision or other manipulations [112]. Since membrane blebs should contain substantially less proteins than plasma membranes prepared by conventional differential centrifugation methods, it was decided to isolate bleb membrane proteins and subject them to proteomics procedures [113]. When crude membrane proteins isolated from blebs were reconstituted into the giant proteoliposomes, they retained the biophysical profile of Maxi-Cl, including selectivity, voltage-dependent inactivation, and sensitivity to Gd^3+^ ions. Preparative liquid phase isoelectric focusing with parallel SDS-PAGE electrophoresis and functional reconstitution allowed us to obtain a fraction that apparently contained only a few dozen visible protein bands. However, the sensitive nano-LC-MS/MS method revealed the presence of over 400 different proteins in this fraction, 93 genes remained in the list after removing enzymes, cytoplasmic components, and unrelated proteins. siRNA screening of the priority list of 15 genes pointed to *Slco2a1* (solute carrier organic anion transporter family member 2A1) as a possible candidate. Gene silencing targeting four different sites (two using siRNA and two by microRNA) produced comparable suppression of the channel activity, excluding possible off-targeting artefacts. Moreover, currents could be restored in stable microRNA-transfected cells by a microRNA-insensitive variant of SLCO2A1. HEK293T cells do not express SLCO2A1 protein and have no Maxi-Cl activity. Heterologous expression of the cloned mouse *Slco2a1* gene in these cells led to the appearance of Maxi-Cl-like channel activity. Furthermore, purified recombinant SLCO2A1 proteins, when reconstituted into the giant proteoliposomes, produced Maxi-Cl channel activity, which was reversibly blocked by Gd^3+^ ions. SLCO2A1 is known as a prostaglandin transporter (PGT) [114]. A PGT substrate, prostaglandin E2 (PGE2), measurably blocked the Maxi-Cl channel amplitude in C127 cells, whereas a PGT blocker, bromosulfophthalein (BSP), produced a potent flickery block of the Maxi-Cl channel activity with *IC_50_* close to that for PGT inhibition (Figure 4). Charge-neutralizing K613G mutation is known to impair the PGT function of SLCO2A1 [115]. This mutation decreased the channel amplitude and conferred a weak cationic selectivity to the heterologously expressed SLCO2A1 in HEK293T cells, as well as to the recombinant channel reconstituted into the giant proteoliposomes. Overexpression of K613G and R560N (another charge-neutralizing and PGT-disrupting mutant) led to a significant decrease in channel amplitude and shifted its voltage dependency. Two other disease-causing SLCO2A1 mutants, G222R and P219L, which are associated with pachydermoperiostosis in humans [116,117], were non-functional in the channel activity. These lines of evidence strongly suggest that SLCO2A1 constitutes the core component of the Maxi-Cl channel.

Regulated conductive ATP release is attributed to the Maxi-Cl activity as its major physiological function [13,18,22,23,33,34,35,36]. Consistent with this notion, the swelling-induced release of ATP from C127 cells was markedly suppressed by an SLCO2A1 blocker BSP and by *Slco2a1* gene silencing by specific siRNA, whereas a minute endogenous ATP release from HEK293T cells was largely augmented by heterologous expression of the SLCO2A1 protein [113]. In the isolated Langendorff-perfused mouse heart model, massive release of ATP was observed upon re-perfusion after brief episodes of oxygen-glucose deprivation, and this ischemia/reperfusion-associated ATP release was markedly suppressed by in vivo silencing the *Slco2a1* expression achieved by tail vein injections of specific siRNA [113].

Interestingly, although CRISPR/Cas9-mediated *Slco2a1* gene knock-out abolished the Maxi-Cl phenotype in C127 cells [113], some residual channel activity with non-Maxi-Cl phenotype (lower amplitude and weaker anion-to-cation selectivity) still could be recorded. It was supposed that SLCO2A1 knockout may upregulate some *Slco2a1* paralogs, or other subunits of the multicomponent native Maxi-Cl, orphaned upon gene knockout, may combine with different membrane proteins to result in channels with a different phenotype. It is also possible that this channel activity, which was PGE2-insensitive, could be associated with plasmalemmal VDAC protein mentioned above.

The function of SLCO2A1 as a transporter for prostaglandins is well characterized [114,118,119,120,121]. We supposed that this function is mainly used for the resting state when the protein itself or its regulatory component(s) is (are) phosphorylated. Upon stimulation, the protein undergoes a conformational change and acquires an ion channel function. Thus, SLCO2A1 can be added to the class of bimodal channel/transporter proteins [122], which comprises TMEM16 family members, both Ca^2+^-activated Cl^−^ channels and lipid scramblases [123]; ClC family proteins known to function as chloride channels and Cl^−^/H^+^ exchangers [124,125,126]; excitatory amino acid transporter (EAAT) proteins of the SLC1A family providing a channel pathway for anions within the same molecule [127]; and SLC26A3/6 serving as chloride/bicarbonate exchangers or Cl^−^ channels depending on ionic conditions [128].

## 6. Molecular Identity of Maxi-Cl: The Regulatory Components

**Figure 5:**
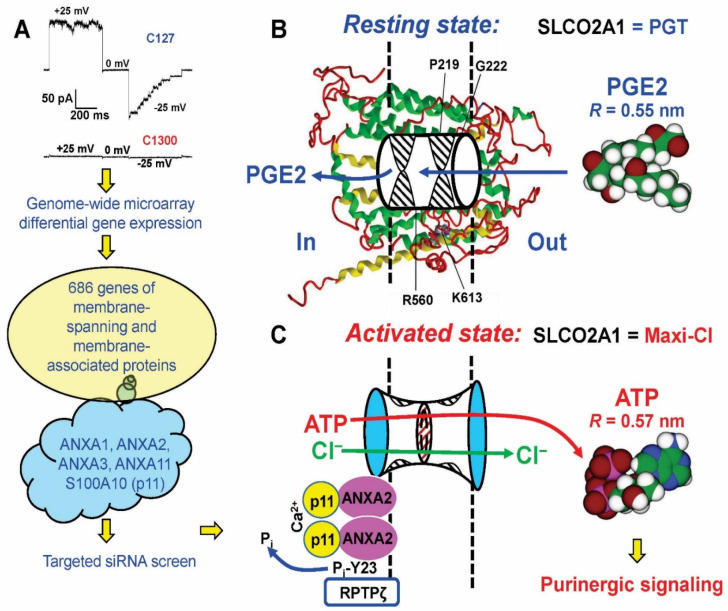
Regulatory components of Maxi-Cl and its bimodal function as a channel and a transporter. (**A**) Steps in identification of ANXA2 and S100A10 (p11) as regulatory components of Maxi-Cl. The scheme was modelled after [91]. See text for details. (**B**) Function of SLCO2A1 as a prostaglandin transporter (PGT) in the resting state. The background molecular model was adopted from [113]. Disease-related mutations are indicated. The cylinder with two hypothetical gates (hatched) denotes a path for PGE2 uptake. (**C**) Function of SLCO2A1 as a Maxi-Cl channel in the activated state. The sizes of two vestibules and of the selectivity filter are same as in Figure 3B. ANXA2 and p11 (S100A10) are known to form a heterotetramer, which supposedly binds to the intracellular surface of the lipid matrix and interacts with the channel core. Ca^2+^ binding at p11 and dephosphorylation at the Tyr23 residue of ANXA2 (by RPTPζ and some other tyrosine phosphatases) are prerequisite events for channel activation. The radii of PGE2 and ATP are calculated as geometric means of three dimensions using Molecular Modeling Pro software (Norgwyn Montgomery Software Inc., North Wales, PA, USA); the molecules are shown bigger compared to the pore, not in scale. For in-scale relationship see Figure 3B.

The native Maxi-Cl channel is inactive in resting cells and is activated upon various physiologically meaningful stimuli or patch excision [33]. The channel activation involves dephosphorylation of tyrosine residues by receptor protein tyrosine phosphatases, including RPTPζ [62], as was mentioned above. Since native Maxi-Cl in blebs and recombinant channels in proteoliposomes were in constitutively active states [113], it is conceivable that native Maxi-Cl is a multicomponent complex containing a core pore-forming SLCO2A1 protein and some auxiliary regulatory components. Mouse neuroblastoma C1300 cells were reported previously to express maxi-anion channels, which are activated in response to antiestrogens, tamoxifen, and toremifene [85]. However, the variant of this cell line used in our experiments did not exhibit any MAC activity upon patch excision or prolonged treatment with tamoxifen (Figure 5A). Thus, these cells were used as a Maxi-Cl-deficient counterparts of the channel-rich mouse C127 cells for further analysis of differential gene expression at the mRNA level performed by a genome-wide microarray technique, and the following results were obtained by Islam et al. [91]. Such differential gene expression procedures resulted in a total of 686 potential membrane-spanning or membrane-associated proteins highly expressed in C127, but not in C1300 cell lines. Consistent with a previous study [113], the SLCO2A1 mRNA showed the highest signal ratio, confirming our conclusion that this protein constitutes the core of Maxi-Cl. The list of candidate proteins found in the membrane blebs [113] and differentially expressed in C127/C1300 cells contained four members of the annexin family, ANXA1, ANXA2, ANXA3, and ANXA11 (Figure 5A). Annexins are known to be Ca^2+^-dependent phospholipid-binding proteins involved in numerous cellular functions. When we silenced these four annexins and also ANXA6, which has been previously attributed to the MAC regulation in placental syncytiotrophoblasts [129], only *Anxa2* gene silencing resulted in reduced Maxi-Cl activity. ANXA2 protein colocalized with SLCO2A1 and even co-precipitated with this protein, indicating a close physical interaction between the two proteins. Overexpression of ANXA2 alone in C1300 cells did not produce any channel activity, whereas SLCO2A1 expression produced BSP-sensitive Maxi-Cl-like currents which, in turn, were significantly augmented by coexpression with ANXA2. ANXA2 is known to be phosphorylated at Tyr23 by protein tyrosine kinases. Overexpression of the phosphorylation-mimicking mutant *Anxa2*-Y23E led to a dramatic suppression of the Maxi-Cl activity, clearly suggesting that Tyr23 plays a critical role in channel activation. ANXA2, which belongs to Ca^2+^-binding proteins with two consecutive EF-hands, is known to form a heterotetrameric complex with S100A10 protein (also known as p11) [130]. In fact, S100A10 was found to show differential C127/C1300 expression (Figure 5A). The Maxi-Cl activity was sensitive to knockdown of this protein expression by siRNA and was prominently inhibited by the interfering synthetic peptide, Ac-(1–14), which disrupts the ANXA2-S100A10 interaction. This result strongly suggests that ANXA2/S100A10 complex formation is essential for Maxi-Cl activity. ANXA2 itself is Ca^2+^-dependent, while complexing with S100A10 makes this dependency even stronger [131]. Maxi-Cl in excised inside-out patches is activated by cytosolic Ca^2+^ with *K_d_* of 0.5 µM, and this activation by Ca^2+^ was almost abolished by siRNA-mediated knockdown of S100A10 expression, suggesting that this protein, often called an annexin light chain, is in fact responsible for Ca^2+^ dependency of Maxi-Cl that was previously demonstrated by channel activation by application of Ca^2+^ ionophore [66,78,79,88]. Based on the above-mentioned experimental observations, Islam et al. [91] concluded that the SLCO2A1 core of Maxi-Cl forms the channel pore; the channel is kept in the closed state by ANXA2 phosphorylated at its Tyr23 and can be activated by dephosphorylation of this residue; and channel activity requires binding of Ca^2+^ ions at the EF-hands motifs of S100A10 and, possibly, also at the non-EF Ca^2+^-binding sites of ANXA2. The cartoon in Figure 5B,C illustrates the bimodal feature of the SLCO2A1 protein, which functions as a prostaglandin transporter in the resting state and turns to the Maxi-Cl channel in the activated state.

It should be noted that the ANXA2-S100A10 complex has been implicated in regulation of other ion channels, including volume-sensitive outwardly rectifying (VSOR/VRAC) anion channel [132] and CFTR [133,134,135].

## 7. Physiological/Pathophysiological Implications of Maxi-Cl

As a swelling-activated chloride channel, Maxi-Cl is implicated in cell volume regulation as an efficient pathway for Cl^−^ in KCl efflux during the regulatory volume decrease (RVD) upon hypoosmotic stress [63,73,74,75,82,136,137,138,139,140]. The Maxi-Cl channel is suggested to provide a charge balance in the apoptotic volume decrease (AVD) during apoptosis [105], potassium uptake in Schwann cells [141,142], H^+^ translocation in Golgi complex [143], and electron transfer by NADPH oxidase in B cells [87]. Chloride movements in fluid secretion during humor formation in eyes [144,145] and hepatic bile ducts [89,90] were suggested to occur via Maxi-Cl. The channel bicarbonate permeability is thought to be important for its function in pancreatic duct cells [146], in alveolar epithelium [147], in placentas [129,148], and in carotid bodies [149]. As a channel permeable to small organic anions, Maxi-Cl is suggested as one of the pathways for transport of short chain fatty acids, such as acetate, propionate, and butyrate, in the gastrointestinal tract of ruminants [48,49,50,51].

Since the Maxi-Cl channel is permeable to nucleotides ATP, ADP, and UTP, its activation triggers physiologically and pathophysiological very important purinergic signaling cascades. Purinergic cell-to-cell signal transduction is an important event in normal cardiac and brain functions and has been greatly reinforced in pathological situations such as ischemia, trauma, stroke, inflammation, and tumor-host interactions [10,150,151]. ATP release in association with Maxi-Cl activation was confirmed in mammary C127 cells [54,91,113], in L929 fibrosarcoma cells [55] in response to osmotic swelling, in kidney macula densa cells in response to salt stress [56], in cardiomyocytes in response to ischemia, hypoxia and osmotic stress [57,58], in cultured astrocytes in response to oxygen/glucose deprivation [60] and osmotic swelling [152], and in isolated Langendorff-perfused adult mouse hearts in response to ischemia/reperfusion [113,153].

The swelling-induced rate of ATP release from osmotically swollen cardiac myocytes was estimated to be around 1.1 × 10^5^ molecules s^–1^ cell^–1^ [57]. Considering the single-channel amplitude of –20 pA at –50 mV, we arrive at the transport rate of 12.5 × 10^7^ elementary charges per second. If we assume that the intracellular ATP concentration is 1/10 of that for Cl^−^ (e.g., 2 mM ATP and 20 mM Cl^−^, respectively) and the permeability ratio is *P_ATP_/P_Cl_* ~0.1, we can estimate that a single fully open Maxi-Cl channel transports about 3 × 10^5^ molecules of ATP^4–^ per second at –50 mV or twice as many MgATP^2–^ molecules. This rate suggests that a brief opening of only several Maxi-Cl channels would be sufficient to provide the observed rate of ATP release. Since a single excised inside-out patch often contains up to 10–15 Maxi-Cl channels, it can be concluded that a single cardiac myocyte possesses a quite sufficient number of ATP-releasing maxi-anion channels to generate adequate physiological or pathophysiological responses. We believe that this inference is valid also for other Maxi-Cl-expressing cells.

In addition to ATP and other nucleotides, Maxi-Cl openings lead to the release of other signaling molecules. Thus, release of glutamate in response to osmotic stress and chemical ischemia was shown to occur partially via Maxi-Cl in primary cultured astrocytes [59]. Another physiologically important signaling molecule is glutathione, with an effective radius of 0.52–0.56 nm, which is well below the size of the Maxi-Cl pore and is close to the pore dimensions of VSOR [154]. Rat thymocytes were shown to massively release GSH in response to osmotic swelling, which was, however, insensitive to the Maxi-Cl blocker, Gd^3+^ [71]. Functional expression of Maxi-Cl was demonstrated for mouse thymocytes [63], however no Maxi-Cl activity was detected in thymocytes from rats (Kurbannazarova, Kurganov and Sabirov, unpublished observation), which is consistent with the fact that no contribution of this channel to the swelling-induced GSH release from rat thymic cells has been found. Since Maxi-Cl is permeable to GSH (see Figure 2B,D), the biophysical and pharmacological profile of glutathione release from other Maxi-Cl expressing cells (such as cardiac myocytes and glia) needs to be explored.

Molecular identification of the core component of Maxi-Cl as a protein encoded by the *Slco2a1* gene opens up perspectives to relate the channel to human diseases. In a recent comprehensive review, Nakanishi et al. [155] listed 83 mutations in coding regions and 15 mutations in intronic regions of the human *SLCO2A1* gene clinically associated with two human diseases: primary hypertrophic osteoarthropathy (PHO), which is also called pachydermoperiostosis (PDP), and chronic enteropathy, which is associated with *SLCO2A1* (CEAS). Mutations (missense, nonsense, deletions, and frame shifts) are spread throughout the gene and were found in each of the 12 transmembrane helices, as well as in the exra- and intracellular loops, and even at the start codon (see Table 4 and Figure 4 in [155]). Although there were differences in age of onset and occurrence in males and females, clinical manifestations characteristic of PHO, such as pachydermia (abnormal thickening of the facial and head skin), periostosis (abnormal formation of periosteal bone), and finger clubbing, were found together with features of CEAS, such as chronic bleeding with multiple small-intestinal ulcers [156,157,158,159]. Mutations, such as G222R, G255E, and P219L, were suggested to affect the transport function of SLCO2A1 protein based on protein modelling [116,117]. Also, a nonsense mutation G104X was shown to be coupled to familial digital clubbing, colon neoplasia, and NSAID resistance [160]. In in vitro studies, mutations G222R, R603X, E141X, V458F, and G183R, as well as c.940+1G>A (a splice site change), led to impairment of prostaglandin transport in SLCO2A1-transfected HEK293 cells [156]. A similar effect was also shown for the L563P mutant [161]. These results suggest that PHO and CEAS pathogenesis is related to aberrant plasma membrane prostaglandin transport, which normally keeps the extracellular PGE2 levels low, and its failure results in augmented extracellular PGE2 and, consequently, in chronic inflammation at the affected sites. Since two disease-causing mutants, G222R and P219L, resulted in the loss of the Maxi-Cl channel function in the HEK293 overexpression experiments [113], we suppose that impairment of the ion channel function of the SLCO2A1 protein could be involved in the pathogenesis of said diseases. The underlying mechanisms remain enigmatic and could encompass channel-mediated purinergic signaling and glutathione-related pathways. The established function of the maxi-anion channels in transport of short-chain fatty acids [48,49,50,51] may provide a rational for the channel-mediated mechanism of pathogenesis of the intestine.

A number of other pathologies have also been associated with SLCO2A1. Thus, recent studies have provided new evidence for the roles of the SLCO2A1 protein in carcinogenesis [160,162,163,164,165,166], in experimental colitis [167], in the parturition process [168], in cigarette-smoke-induced lung inflammation [169], in a gastroesophageal reflux disease [170], and in the non-healing diabetic foot ulcer [171]. The role of the ion channel function of SLCO2A1 in the mechanism of development of these pathologies remains to be elucidated.

## 8. Concluding Remarks

The maxi-anion channels represent a heterogeneous population of ion-transporting pathways in which the phenotype of Maxi-Cl with distinct biophysical profile accounts for a majority of reports. Identification of Maxi-Cl as a complex with SLCO2A1 as a core pore-forming component and two auxiliary proteins, annexin A2 providing the substrate site for tyrosine kinases and phosphatases and S100A10 conferring Ca^2+^ sensitivity, provided mechanistic grounds to the role of Maxi-Cl as one of the major pathways for regulated effluxes of signaling molecules, ATP^4–^, MgATP^2–^, UTP, glutamate, and, possibly, glutathione. The relationship with the prostaglandin transporter could be somewhat confusing. However, more lines of evidence are accumulating that suggest that nature uses bimodality to achieve a complex balance of numerous metabolic and signaling pathways in order to maintain reliable functioning of life machinery. SLCO2A1 malfunctioning leads to human diseases, and discovering of the fine molecular PGT- and Maxi-Cl-involving mechanisms of pathogenesis is an enthralling task. SLCO2A1 in both transporter and channel modes is expected to serve as a perspective pharmacological target for developing new therapeutics for treatment of both inherited and acquired skin, bone, gut, neuronal, and muscular disorders, as well as malignancies involving SLCO2A1.
